# Whole genome sequencing and tuberculosis surveillance and control: a critical view from resources limited settings

**DOI:** 10.3389/fpubh.2026.1975587

**Published:** 2026-10-21

**Authors:** Ángel Sebastián Rodríguez-Pazmiño, Solon Alberto Orlando, Greta Franco Sotomayor, Miguel Angel Garcia-Bereguiain

**Affiliations:** 1 One Health Research Group, Universidad de Las Américas, Quito, Ecuador; 2 Universidad Espíritu Santo, Samborondón, Ecuador; 3 Instituto Nacional de Salud Pública e Investigación, Guayaquil, Ecuador; 4 Universidad Católica Santiago de Guayaquil, Guayaquil, Ecuador

**Keywords:** Ecuador, End TB Strategy, surveillance, tuberculosis, WGS–whole-genome sequencing

## Introduction

Whole-genome sequencing (WGS) is increasingly presented as a transformative tool for tuberculosis (TB) surveillance, drug-resistance detection, and precision treatment (1, 2). Its scientific value is not in dispute. As countries work toward the milestones of the WHO End TB Strategy (3), however, an important question for resource-limited settings is not whether genomic tools work, but where they should sit among competing priorities. In this opinion piece, using Ecuador as a case study, we argue that genomic sequencing is a legitimate and useful technology that should continue to be developed locally, but that it should not be treated as the central lever for ending the TB epidemic where the foundations of case detection, treatment retention, and social protection remain fragile.

We frame this deliberately as a question of sequencing priorities rather than a choice between technology and equity. The End TB Strategy already integrates early diagnosis and treatment, social protection, action on social determinants, universal health coverage, and research and innovation as mutually reinforcing pillars (World Health Organization, 2015). Our concern is with the order and proportion in which a country such as Ecuador, under severe fiscal constraint, invests in them.

## A rising and unevenly distributed burden

Tuberculosis in Ecuador is not a receding problem. According to the WHO Global Tuberculosis Report 2025, the country's estimated TB incidence rate rose by approximately 57% between 2015 and 2024, and estimated TB mortality increased by about 34% over the same period (4). WHO also emphasizes that a substantial share of incident TB in the Region of the Americas is never diagnosed or notified, so official counts likely understate the true burden (4). Although Ecuador is not among WHO's 30 high-burden countries, its trajectory is deteriorating rather than stable (5).

Crucially, this burden is highly concentrated. National surveillance data indicate that people deprived of liberty accounted for close to one quarter of notified TB cases in 2025 (6). Part of this rise reflects intensified molecular screening that reached most of the incarcerated population; yet even with improved case-finding, the concentration remains remarkable.

An earlier study in Ecuador's largest men's prison estimated a TB incidence of roughly 3,947 per 100,000 in 2015–2016, orders of magnitude above the general population (7). Provincial notification data further show the epidemic clustering along impoverished coastal corridors such as El Oro and Guayas (6). These are settings shaped by overcrowding, poverty, and interrupted access to care—the classic social determinants of TB.

## Where the care pathway is weakest

The most important prioritization consideration is where Ecuador's TB response loses ground. Retention in care for drug-resistant TB has been a long-standing weakness: in a national cohort of patients with rifampicin-resistant or multidrug-resistant TB treated in 2014–2015 under the former 18–24-month regimen, loss to follow-up reached 39.6% and treatment success was 50.4% (8). Shorter, better-tolerated all-oral regimens have since been introduced and may have improved these outcomes (4); updated national figures are needed. Even so, loss to follow-up on this scale reflects structural, financial, and geographic barriers patients face rather than individual failure, and it identifies the part of the system most in need of investment.

This bears directly on the genomics debate. Comprehensive resistance profiling is only as useful as the system's capacity to act on it—to reach patients, begin an effective regimen quickly, and support them to completion. Where many patients disengage from care, the marginal benefit of higher-resolution resistance data is constrained by the same structural gaps that drive the epidemic.

## What the evidence supports, and where

The economic case for routine WGS is strongest in high-income, low-burden settings such as the United Kingdom, where health systems already ensure prompt diagnosis and high treatment completion, so sequencing can deliver measurable transmission and cost benefits (9). Implementation in lower-resource, high-burden settings is feasible—as shown in the Kyrgyz Republic—but those experiences also document the substantial equipment, reagent, training, and quality-assurance costs involved (2, 10). The living conditions and system performance that make sequencing cost-effective in the United Kingdom are precisely what Ecuador lacks.

By contrast, interventions addressing the social determinants of TB have a strong and growing evidence base in Latin America. In Peru, the CRESIPT randomized controlled trial showed that socioeconomic support improved treatment success and uptake of preventive therapy (11). In Brazil, participation in a conditional cash-transfer program was associated with substantially lower TB incidence and mortality in the 100 Million Brazilian Cohort (12). A case-control study in Peru estimated that about 47% of TB was attributable to poverty—a figure from a comparable Andean setting that, while not directly transferable to Ecuador, conveys the scale of the opportunity (13).

We do not claim a simple dollar-for-dollar trade-off between a sequencer and a food basket; TB budgets are not perfectly fungible, and genomic capacity is often financed through research grants or multi-pathogen surveillance funds. But all programs ultimately compete for finite public-health resources and political attention, and transparent priority-setting should follow the evidence of what most reduces TB in a given context.

## The role of sequencing: legitimate, but not a panacea

None of this argues against genomic tools. WGS has clear value for resolving transmission clusters, detecting second-line drug resistance when conventional testing is inconclusive, and advancing research; WHO now recommends targeted next-generation sequencing (tNGS) as a follow-on drug-resistance test in specific clinical situations (14). WGS and tNGS are not interchangeable, and current WHO guidance reserves them for defined uses rather than universal application. Ecuador should continue to build this capacity.

The published Ecuadorian WGS literature nevertheless remains limited and largely exploratory: studies to date have characterized modest numbers of isolates—for example, 24 strains (21 passing quality control) in one analysis and 88 in another—or compared bioinformatics tools, rather than sustaining operational surveillance (15–17). Turning this into a durable public-health tool will require local workforce, computing infrastructure, and data governance. Where these are underdeveloped, sequencing may come to depend on external analysis and funding; this is a governance challenge to be managed through capacity-building and equitable data arrangements, not an inevitable feature of the technology (10, 18).

## Discussion

Several reasonable counterarguments deserve acknowledgment. Genomic surveillance could, in principle, reveal unrecognized prison-to-community transmission and help target interventions in exactly the high-burden settings we highlight; comprehensive resistance profiling can shorten the time to an effective regimen and may thereby support retention; and centralized or sentinel sequencing can add value without sequencing every notified case. Retention in care and genomic testing are therefore not wholly independent. These points strengthen, rather than weaken, the case for a deliberate, criteria-based approach: expanding sequencing where a clear public-health use case, adequate specimen referral, quality assurance, trained personnel, sustainable financing, and a defined pathway from result to action are in place—while prioritizing the foundations elsewhere.

## Conclusion

For Ecuador and countries with similar profiles, we suggest a staged approach. In the near term, sequencing is best deployed selectively—for outbreak and prison-cluster investigation, second-line resistance confirmation, and sentinel drug-resistance surveillance—while the largest new investments go to case-finding in prisons and poor communities, retention support, and social protection. Routine or universal WGS can be revisited as diagnostic coverage, treatment completion, and laboratory and bioinformatics capacity improve, tracked with indicators such as time to effective therapy and cost per actionable result. Precision medicine ultimately delivers value only when it changes what happens to the patient. In Ecuador today, the most decisive gains against TB lie in strengthening the social and health-system foundations on which any genomic tool must stand.
